# Ginsenoside from ginseng: a promising treatment for inflammatory bowel disease

**DOI:** 10.1007/s43440-020-00213-z

**Published:** 2021-01-19

**Authors:** Zengping Kang, Youbao Zhonga, Tiantian Wu, Jiaqi Huang, Haimei Zhao, Duanyong Liu

**Affiliations:** 1 Graduate School, Jiangxi University of Traditional Chinese Medicine, Nanchang, 330004 Jiangxi China; 2 Experimental Animal Science and Technology Center, Jiangxi University of Traditional Chinese Medicine, Nanchang, 330004 Jiangxi China; 3 College of Traditional Chinese Medicine, Jiangxi University of Traditional Chinese Medicine, 1688 Meiling Road, Nanchang, 330004 Jiangxi China; 4 Science and Technology College, Jiangxi University of Traditional Chinese Medicine, 1689 Meiling Road, Nanchang, 330004 Jiangxi China

**Keywords:** Ginsenosides, Immune cells, Inflammatory bowel disease, Pharmacological effects

## Abstract

Inflammatory bowel disease (IBD) is an autoimmune disease mediated by immune disorder and termed as one of the most refractory diseases by the Word Health Organization. Its morbidity has increased steadily over the past half century worldwide. Environmental, genetic, infectious, and immune factors are integral to the pathogenesis of IBD. Commonly known as the king of herbs, ginseng has been consumed in many countries for the past 2000 years. Its active ingredient ginsenosides, as the most prominent saponins of ginseng, have a wide range of pharmacological effects. Recent studies have confirmed that the active components of Panax ginseng have anti-inflammatory and immunomodulatory effects on IBD, including regulating the balance of immune cells, inhibiting the expression of cytokines, as well as activating Toll-like receptor 4, Nuclear factor-kappa B (NF-κB), nucleotide-binding oligomerization domain-like receptor (NLRP), mitogen-activated protein kinase signaling, and so on. Accumulated evidence indicates that ginsenosides may serve as a potential novel therapeutic drug or health product additive in IBD prevention and treatment in the future.

## Introduction

Inflammatory bowel disease (IBD) is a chronic inflammatory intestinal disease with unknown etiologies and pathogenesis, including ulcerative colitis (UC) and Crohn's disease (CD). The visible clinical symptoms of patients with IBD are abdominal pain, diarrhea, and rectal bleeding [1]. According to the World Health Organization, IBD is one of the most refractory diseases around the world. The incidence and death rates for IBD have increased over the last half century, especially in newly industrialized countries [2–4]. Many investigations showed that psychological disorders and malnutrition are observed in patients with IBD, which not only increase the economic burden on patients but also impact negatively on their quality of life [5]. Currently, 25% of drugs used to treat IBD are made from herbs, while 10% are made from microbial sources. As the king of all herbs, ginsenosides, the main active components of Panax ginseng belonging to the family Araliaceae, have various biological and pharmacological effects, such as anti-inflammatory, immunomodulatory, antioxidant, and others. Since ancient times, ginsenosides have been used as food additives in soups and tea drinks in Southeast Asian countries, such as China and have developed into functional foods to prevent inflammation. Previous in vitro and in vivo studies demonstrated that the pharmacological effects of ginsenoside on IBD involved the regulation of immune cell differentiation, cytokine secretion, and inflammatory signal activation. Recent studies focused on the role of natural compound ginsenoside in treating IBD. The findings indicated that the potential therapeutic effects of ginsenosides on IBD were partially mediated by regulating the balance of immune status, cytokine expression, and activation of inflammation-related signaling pathways.

## Ginsenosides

**Fig. 1:**
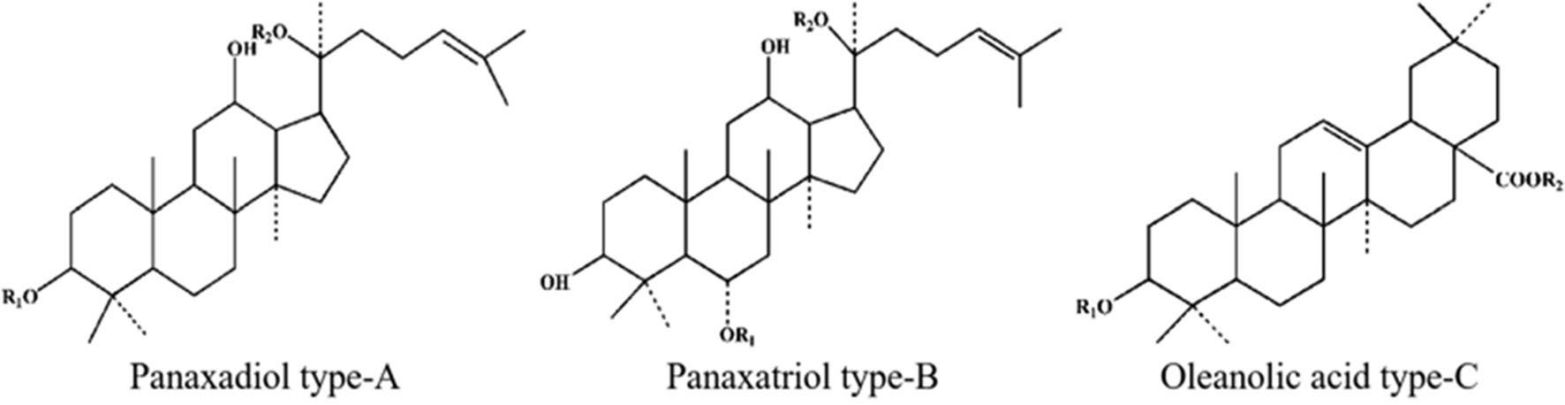
Schematic diagram of chemical structure of ginsenosides

Ginseng, the dried root or rhizome of Panax ginseng belonging to the family Araliaceae, was first referred to in the book Sheng Nong's *Herbal Classic* [6]. In China, it is known as the "king of all herbs." In the West, it is called Panax ginseng C.A. Meyer, "Panax," which is a Greek word meaning "cure all diseases." Noteworthily, ginsenosides are the main active components of ginseng herbal medicine. They are extracted from roots, stems, leaves, flowers, and fruits of ginseng. They have pharmacological properties, including anti-inflammatory, antitumor, antifibrotic, and glucose lowering [7]. Ginsenosides are polysaccharide derivatives mainly comprising semi-acetal hydroxyl groups of sugars and nonsugar compounds. More than 100 kinds of ginsenosides have been isolated and identified to date [7]. Ginsenosides have a similar chemical structure and are composed of triterpenoid saponins with 30 carbon atoms. They have three main categories based on the structural differences: A, B, and C (Fig. 1). Type A (20 (s)-protopanaxadiol-type saponins] and type B [20 (s)-protopanaxatriol-type saponins) are dammarane-type tetracyclic triterpenoid saponins; most ginsenosides belong to this kind of saponins. C-type saponins are oleanane-type pentacyclic triterpenoid saponins, such as ginsenoside R, which are relatively common in the nature (Table 1).

**Table 1:** Classification of ginsenosides

Types	Ginsenoside	R1	R2	Formula	Molecular mass	References
Panaxadiol type A	Ginsenoside Rb1	glc(2–1)glc	glc(6–1)glc	C54H92O23	1108	[59]
	Ginsenoside Rb2	glc(2–1)glc	glc(6–1)ara(p)	C53H90O22	1078	[59]
	Ginsenoside Rb3	glc(2–1)glc	glc(6–1)xyl	C53H90O22	1078	[60]
	Ginsenoside Rc	glc(2–1)glc	glc(6–1)ara(f)	C53H90O22	1078	[59]
	Ginsenoside Rd	glc(2–1)glc	glc	C48H82O18	946	[59–61]
	Ginsenoside Rg3	glc(2–1)glc	H	C42H72O13	784	[62, 63]
	Ginsenoside Rh2	Glc	H	C36H62O8	622	[63]
Panaxatriol type B	Ginsenoside Re	glc(2–1)rha	glc	C48H82O18	946	[61, 62]
	Ginsenoside Rf	glc(2–1)glc	H	C42H72O14	800	[62]
	Ginsenoside Rg1	Glc	glc	C42H72O14	800	[62]
	Ginsenoside Rg2	glc(2–1)glc	H	C42H72O13	784	[63]
	Ginsenoside Rh1	Glc	H	C36H62O9	638	[63]
Oleanolic acid type C	Ginsenoside Ro	glcUA(2–1)glc	glc	C48H76O19	956	[59]

*glc* β-d-glucopyranoside, *ara(p)* arabinopyranoside, *ara(f)* furanoside, *xyl* xylose group, *rha* rhamnose

### Pharmacological effects of ginsenosides

For more than 2000 years, ginseng has been regarded in traditional Chinese medicine as a panacea for prolonging life, which can effectively relieve mental stress and physical fatigue [8]. It is not only widely used in the clinic, but also consumed as food materials or additives, health products, and other functional foods in daily life, such as ginseng wine, preserved ginseng fruit, ginseng biscuits, ginseng essence oral liquid, and other products. Sun et al. confirmed that the long-term intake of ginsenosides from the ginseng extract could promote the production of intestinal probiotics and the secretion of anti-inflammatory factors (IL-4 and IL-10), and immunoglobulin (Ig) A (IgA), by nontargeted gas chromatography with time-of-flight mass spectrometry (GC-TOFMS) metabonomics analysis of serum, cecum, and ileum contents [9]. These results showed that long-term administration of ginsenosides had positive effects on host intestinal metabolism, immunity, and intestinal flora balance. With the scientific and technological progress and commodity globalization, ginseng is widely used all over the world. The edible and medicinal values of ginseng have been one of the hotspots of the current new drug research and development because ginsenosides have a variety of pharmacological activities, such as antitumor, anti-inflammatory, analgesic, and antiaging, besides regulating immune homeostasis.

A large body of evidence shows that some monomer components of ginsenosides have protective effects in multiple organs, tissues, and systems (Table 2). Ginsenosides protect against arrhythmia, myocardial hypertrophy, cardiomyocyte apoptosis, myocardial ischemia–reperfusion injury, and heart failure in the cardiovascular system. It can be used to treat neurodegenerative diseases, improve memory function, protect brain tissue, regulate metabolism, treat diabetes and obesity, and regulate insulin levels. Also, it can effectively prevent and control lung cancer, esophageal cancer, gastric cancer, liver cancer, and breast cancer by inducing cancer cell apoptosis and inhibiting cancer cell proliferation. In addition, ginsenosides also have many other effects, such as whitening [10], relieving itching [11], anti-inflammation [12], antivirus [13], and regulating immunity. Among these, the role of ginsenosides in maintaining immune homeostasis is directly or indirectly realized by extensive and clear regulation of immune cells (Table 3). The immunomodulatory effect of ginsenosides is critical in the process of IBD treatment.

**Table 2:** Modern pharmacological effects of ginsenosides

Ginsenoside	Cardiovascular system	Nervous system	Regulate metabolism	Antitumor effect	References
Ginsenoside Rb1	Cardiomyocyte apoptosis, arrhythmia, and vascular calcification	Stroke, memory impairment, neuro degenerative diseases	Obesity, hyperglycemia, diabetes	Uterine leiomyoma and ovarian cancer	[64–74]
Ginsenoside Rb2	Not reported	Neurotoxicity	Obesity	Colorectal cancer	[75–79]
Ginsenoside Rb3	Heart failure and myocardial ischemia–reperfusion injury	Not reported	Liver gluconeogenesis	Not reported	[80–82]
Ginsenoside Rc	Not reported	Not reported	Obesity and diabetes	Ovarian cancer	[83, 84]
Ginsenoside Rd	Cardiac hypertrophy	Ischemic stroke	Obesity	Nonsmall cell lung cancer, colorectal cancer	[85–88]
Ginsenoside Rg3	Nonsmall cell lung cancer and colorectal cancer	Alzheimer's disease(AD)	Metabolic syndrome	Pancreatic cancer, gastric cancer, breast cancer	[89–96]
Ginsenoside Rh2	Angiogenesis, myocardial fibrosis	Sleep deprivation	Not reported	Breast cancer, lung cancer	[97–101]
Ginsenoside Re	Heart and myocardial fibrosis	AD	Diabetes	Gastric cancer	[102–106]
Ginsenoside Rf	Not reported	Neuropathic pain and AD	Obesity	Not reported	[107–109]
Ginsenoside Rg1	Myocardial dysfunction	Stroke and AD	Insulin resistance, diabetes mellitus	Breast cancer	[110–115]
Ginsenoside Rg2	Not reported	Neurons damage	Obesity	Not reported	[116, 117]
Ginsenoside Rh1	Not reported	Neurons damage	Not reported	Colorectal cancer	[118, 119]
Ginsenoside Ro	Blood clots	Not reported	Not reported	Melanoma	[120, 121]

**Table 3:** Immune effect of ginsenosides

Ginsenoside	T lymphocyte	B lymphocyte	Macrophages	Dendritic cells	Other immune cells	References
Ginsenoside Rg3	Enhance CD4 + CD25 + Foxp3 + Treg cells	Not reported	Promote the phagocytosis of macrophages to bacteria	Immunogenic tumor cell death by inducing DC	Inhibit neutrophil migration	[122–125]
Ginsenoside Rg1	Promote Th1 differentiation of CD4 + T cells and resist candidiasis	Induce IgA production in mouse B fine cells	Regulate innate immune response of macrophages	Activate dendritic cells and act as vaccine adjuvants	Not reported	[126–129]
Ginsenoside Rb1	Inhibit Th1 and Th17 cells and up-regulate Treg cells	Not reported	Enhance phagocytosis of macrophages to bacteria	Inhibit maturation of DCs	Not reported	[130–132]
Ginsenoside CK	Relieve autoimmune arthritis (AA) by inhibiting T cell activation	Downregulate memory B cells in AA rats	Regulate macrophage function by inhibiting β-arrestin2	Regulate the transport of dendritic cells	Not reported	[133–136]

### Pathogenesis of IBD

An intestinal barrier is very important to maintain host health, and is one of the most metabolically dynamic systems. It is the first line of defense against the invasion of potential pathogens and maintaining immunity homeostasis. The intestinal barrier is composed of physical barrier, immune barrier, and biochemical barrier formed by mucopolysaccharides secreted by intestinal epithelial cells and innate and acquired immune cells and mediated by immune mediators; these barriers work in a coordinated manner. IBD is believed to be a chronic and nonspecific intestinal inflammation caused by intestinal mucosal barrier disorder under the combined action of immunity, genetic, infection, and environmental factors [14, 15]. The underdevelopment and damage of the intestinal physical barrier and immune barrier leads to the onset of IBD. Further, the pathogenesis of IBD is closely related to intestinal endothelial cells and intestinal immune cells [dendritic cells (DCs), macrophages, neutrophils, T lymphocytes, and B lymphocytes] and the levels of secreted cytokines.

### Intestinal epithelial cells

The first line of defense of the gastrointestinal tract against antigen invasion is composed of intestinal epithelial cells (IECs); it is located between the lamina propria immune cells and microorganisms in the intestinal lumen. These mature IECs include mucus-secreting goblet cells, hormone-producing Paneth cells, mechano-sensing tuft cells, and nutrient-absorbing enterocytes. These cells participate in antigen presentation and immune response by secreting mucins, antimicrobial peptides (AMPs), and reactive oxygen species (ROS) [16]. Under antigen stimulation, IECs have the potential to secrete cytokines, which not only recruit immune cells to the sites of injured mucosa to participate in immune response but also directly induce the overexpression of inflammatory cytokines (tumor necrosis factor-α, interleukin-1 beta, and IL-6), thus leading to the occurrence and aggravation of IBD. As an indispensable part of the mucosal barrier, IECs play an important role in maintaining the integrity and dynamic balance of the epithelial barrier [17]. When the epithelial cells undergo excessive apoptosis or tight junctions of the gut are damaged, intestinal microorganisms enter the mucosal layer through intestinal leakage, resulting in the continuous stimulation of antigens, massive recruitment of immune cells, and excessive release of inflammatory mediators [18].

### Intestinal immune cells

Intestinal innate immune cells involve macrophages, dendritic cells, lamina propria lymphoid cells, and neutrophils, which can respond quickly to various invasive pathogenic microorganisms. However, when innate immune cells are dysregulated, they secrete large amounts of pro-inflammatory cytokines, causing intestinal tissue damage. M1 macrophages secrete pro-inflammatory cytokines (NF-α, IL-1β, and IL-6), while M2 macrophages secrete anti-inflammatory cytokines (IL-4 and IL-10). When the M1/M2 ratio is severely unbalanced, inflammatory cytokine storms cause inflammatory damage in the gut. In IBD biopsy tissues, macrophages recognize pathogenic microorganisms secreting inflammatory cytokines (IL-1α, IL-1β, and TNF-α), aggravating intestinal inflammation [19]. Therefore, macrophages may be an effective therapeutic approach to alleviate IBD [20]. Dendritic cells, full-time antigen-presenting cells located in the lamina propria, can efficiently process intestinal antigens and present them for T cells to induce Th17 cell differentiation. During the pathogenesis of IBD, DC inhibitors promote the occurrence of enteritis. CD103 + DCs can promote the differentiation of Treg cells and inhibit the inflammatory response, while CX3CR1 + DCs exacerbate the inflammatory response [21]. Innate lymphoid cells directly or indirectly affect macrophage and dendritic cell differentiation by secreting cytokines and other mediators to exert early immune surveillance and immunomodulatory functions [22, 23]. Neutrophils are important in inflammation and tissue damage in IBD. Recent studies suggested that IBD symptoms improved significantly and neutrophils were promoted from N1 to N2 phenotype when extracellular regulated protein kinases (ERK) protein phosphorylation was inhibited [24].

In addition, adaptive immune cells such as T helper (Th) cells (Th1, Th2, Th17, and Th9 cells) and B cells are also critical in IBD. Studies have confirmed that Th cells are directly or indirectly involved in the intestinal immune response of IBD, resulting in the aggravation or relief of intestinal mucosal inflammation. Naive CD4 + T cells are stimulated by exogenous antigens and then differentiate into various subsets, such as Th1, Th2, Th7, Th9, Th10, Th17, and Treg cells [25]. IBD are classified into two types based on the different sources of cytokines in inflammatory tissues of intestinal mucosa: Th1-cell-mediated CD and Th2-cell-mediated UC. In patients with CD, Th1 cells secrete large amounts of TNF-α and IFN-γ; thus, affecting the secretion of TNF-α by macrophages in the gut and thus exacerbating the inflammatory response. Th2 cells in the mucosal tissues of patients with UC secrete a large amount of IL-5 and IL-13; thus, promoting the apoptosis of IECs and destroying the intestinal mucosal barrier. The balance of Th17 and Treg cells is important in the induction and regulation of intestinal inflammation, and efforts are underway to determine their role in IBD [26]. CD4 + T cells differentiate into Th17 cells after activation of the signal transduction and transcriptional activator 3 (STAT3) pathway, thus promoting Th17 cells to secrete excessive IL-17 and aggravating inflammatory response [27]. Zheng et al. found that the number of Foxp3 + Treg cells decreased significantly in patients with UC compared with healthy individuals [28]. Th9 cells are newly discovered effector T cells stimulated by IL-4 and TGF-β through transcription factors, such as PU.1. Interferon regulatory factor 4 secretes IL-9 and hence destroys the intestinal mucosal barrier by inhibiting the proliferation of IECs and downregulating the expression of cell tight junction proteins [29]. Meanwhile, B cells are important effector cells in the acquired immune system, which mediate humoral immunity by secreting antibodies. Likewise, they also supply opsonins for the maturity and functioning of antigen-presenting cells to develop T cells. B cells are involved in the pathogenesis of IBD. B cells promote chemotaxis and migration of neutrophils to the site of tissue inflammation and aggravate inflammatory injury in the peripheral blood of patients with CD, followed by high expression of Toll-like receptor 2 (TLR2) and IL-8 on the surface of B cells, indicating that the immune activity of B cells was enhanced during IBD morbidity [30]. Breg-like cells (IL-33 + Breg) isolated from mice with IBD inhibited the expansion and functioning of immune effector cells and effectively prevented the development of spontaneous colitis in IL-10 − / − mice after adoptive metastasis [31]. These results indicated that adaptive immune cells were the key to the treatment of IBD, and targeting the differentiation of T lymphocytes and B lymphocytes might be an effective strategy for treating IBD.

### Cytokines

Cytokines are small-molecule proteins with a wide range of biological activities. They are produced by immune and nonimmune cells stimulated by antigens, mitogen, or other factors. They are divided into anti-inflammatory cytokines and pro-inflammatory cytokines. The imbalance between them is an important reason for induced intestinal mucosal injury, intestinal barrier dysfunction, and persistent intestinal inflammation. The breakdown of the intestinal barrier attributes the most to the overproduction of pro-inflammatory cytokines, such as TNF-α, IL-1α, and IFN-γ, which are triggered by the activation of the activating protein 1/mitogen-activated protein kinase (AP-1/MAPK) pathway [32]. In vivo studies found that the downregulated activation of IL-1R and TLR effectively reduced chronic colonic inflammatory injury in mice [33], which was closely related to pro-inflammatory cytokines. Similarly, clinical studies showed that retinoic acid maintained intestinal inflammation by upregulating pro-inflammatory cytokines (IL-17 and IFN-γ) [34]. In addition, anti-inflammatory cytokines (IL-10 and TGF-β1) also played an important role in repairing intestinal inflammatory injury. Huber and his workmates reported that IL-10 suppressed the excessive immune response by inhibiting the polarization of Th1 and Th17 cells to alleviate IBD [35]. In vivo and in vitro studies showed that TGF-β1 was a negative regulator of mucosal inflammation. The activation of TGF-β1 led to a decrease in the expression of pro-inflammatory cytokines in mice with colitis or patients with IBD, thus effectively relieving the clinical symptoms of CD [36]. Interestingly, some cytokines, such as IL-33, have dual immunomodulatory effects. Lopetuso and his workmates reported that IL-33 promoted the recovery of acute colitis by inducing miR-320 to stimulate epithelial regeneration and repair in experimental colitis [37]. In contrast, Zhu showed that IL-33 induced DSS-induced colitis in mice by promoting Th2 response and inhibiting Th1 response in the mesenteric lymph node (MLN) [38]. To sum up, the cytokine played a decisive role in the occurrence and pathogenesis of IBD. It also suggested that the balance of cytokines might be an effective target for IBD treatment.

### Effects of ginsenoside on IBD

Clinically, the therapeutic agents for IBD include cyclosporin, corticosteroids, 5-aminosalicylic acid (Mesalamine), mercaptopurine, antitumor necrosis factor (TNF −) monoclonal antibody, azathioprine, and so on. These drugs are expensive and also have obvious side effects and poor tolerance [39]. Yet, natural medicines are cheap, readily available, and highly effective. Notably, relevant studies reported that ginsenosides, the main active components of Panax ginseng, had anti-inflammatory and immunomodulatory effects on IBD, including improving body weight, decreasing disease activity index (DAI), and the index of colonic weight and body weight or colonic length, restoring pathological damage in colonic mucosa, and reducing microscopic, and macroscopic injury scores [40]. In vivo experiments showed that ginsenosides not only promoted the proliferation of intestinal mucosal epithelium, but also regulated the differentiation of immune cells and the secretion of inflammatory mediators; thus, effectively relieving the symptoms of IBD [41]. In vitro cell experiments showed that ginsenosides could effectively inhibit HT-29 cells to secrete pro-inflammatory cytokines (IL-1β, IL-6, and TNF-α) under lipopolysaccharide (LPS) stimulation [6]. The mechanism of ginsenoside treating IBD was reviewed in this study, thus providing the reference for the clinical application of IBD.

### Ginsenosides improved IBD by regulating the balance of immune cells

**Fig. 2:**
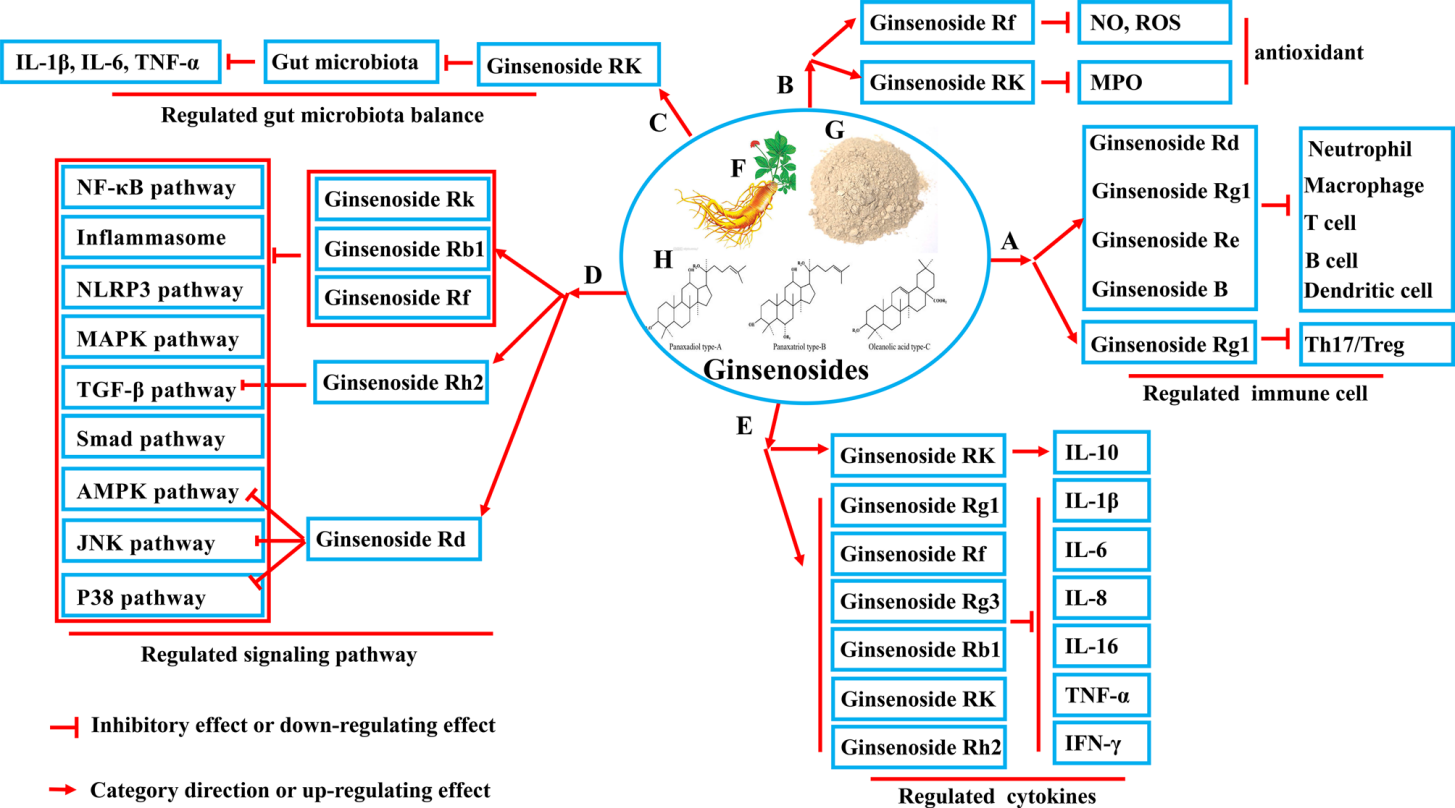
Schematic mechanism of the treatment of inflammatory bowel disease by ginsenosides

With the help of innate immunity, adaptive immunity is activated, and their combined action establishes and maintains the immune homeostasis. As an autoimmune disease, IBD is caused by multiple factors, including environment, genetic predisposition, and immune dysregulation, leading to abnormal differentiation of autoreactive T and B lymphocytes. Yang et al. found that ginsenoside Rd alleviated the symptoms of TNBS-induced animal UC by inhibiting neutrophil infiltration and improving the antioxidant capacity of damaged colon tissue [42]. As shown in Fig. 2a, 20 (S)-protopanaxatriol, Rg1 metabolite of ginsenoside inhibited the binding of TLR4 to LPS on macrophages, restored the balance of Th17/Treg cells, and thus relieved inflammatory diseases such as colitis [43]. Lee et al. used ginsenoside Re to treat TNBS-induced colitis in mice and found that ginsenoside Re could also inhibit the binding of LPS to macrophage membrane TLR4 and further effectively treat inflammation [44]. Moreover, Ginseng berry extract (GB) inhibited the activation of infiltrating T cells, neutrophils, dendritic cells, and macrophages in the colon of mice with DSS-induced colitis and effectively relieved IBD. At the same time, GB promoted the migration of CD103 + CD11c + DCs and the proliferation and differentiation of Foxp3 + Treg cells in the colon of mice with colitis [45]. Fermented red ginseng alleviated TNBS-induced colitis by inhibiting the activation of macrophages and regulating the differentiation of Th1 and Treg cells [46]. To sum up, ginseng and ginsenosides clearly had the potential as an effective target for treating IBD by interfering with the proliferation and differentiation of immune cells.

### Ginsenosides improved IBD by regulating cytokine expression

Both CD and UC are characterized by increased expression of IL-1β, IL-6, IL-8, TNF-α, IL-16, and various T-cell chemokines [47, 48]. As shown in Fig. 2e, ginsenoside Rg1 has been reported to inhibit the release of pro-inflammatory cytokines (IL-1β and TNF-α) by upregulating the expression of NLRP2 and then weaken the inflammatory response of DSS-induced colitis in mice [49]. Ahn et al. showed that ginsenoside Rf significantly decreased the production of IL-1β, IL-6, TNF-α, NO, and ROS in IBD (Fig. 2b), indicating that ginsenoside Rf could treat IBD by inhibiting the expression of inflammatory factors [6]. Some animal experiments by Wang and his workmates showed that ginsenoside Rg3 could significantly ameliorate DSS-induced colitis by inhibiting the expression of pro-inflammatory cytokines (IL-1β and IL-6) [40]. After mice with TNBS-induced colitis were treated by intragastric administration of ginsenoside Rb1, the levels of pro-inflammatory cytokines (TNF-α, IL-1β, and IL-6) declined, while the levels of anti-inflammatory cytokines (IL-10) increased [50]. Li et al. found that ginsenoside metabolite K relieved histopathological damage in mice with DSS-induced colitis, decreased myeloperoxidase (MPO) activity (Fig. 2b), reduced the production of pro-inflammatory cytokines (IL-6, IL-1β, and TNF-α), and increased the production of IL-10 in colon tissue and peripheral blood [51]. Ginsenoside Rh2 significantly decreased the expression of IL-6, TNF-α, and IFN-γ in mice with DSS-induced colitis. Ginsenoside also regulated the levels of inflammatory cytokines; thus, playing a positive role in treating IBD.

### Ginsenosides improved IBD by regulating inflammatory signaling pathway

Many inflammatory signaling pathways are vital in IBD, such as NF-κB, NLRP3, MAPK, AMP-activated kinase (AMPK), and TGF-β/Smad signaling pathway [52, 53]. As shown in Fig. 2d, ginsenoside Rf [44], ginsenoside Rb1 [54], and ginsenoside compound K [55] had regulatory effects on these inflammatory signaling pathways in the process of IBD treatment. Ginsenoside Rd drove autophagy to degrade NLRP3 inflammatory bodies through the AMPK-ULK1-p62 signal axis and downregulated the secretion of IL-1β by inhibiting macrophages to finally treat acute colitis in mice [56]. Furthermore, some studies showed that the protective effect of ginsenoside Rd on TNBS-induced recurrent colitis might be realized by regulating the activation of JNK and p38, reducing leukocyte aggregation, and downregulating the expression of TNF-α, IL-1β, IL-6, and other pro-inflammatory cytokines [57]. The TGF-β signal is considered to be one of the essential anti-inflammatory signaling pathways. Ginsenoside Rh2 may increase the phosphorylation of downstream small mother against decapentaplegic (Smad) signaling by activating the TGF-β signaling pathway, inhibit the activation of pro-inflammatory signal pathways, such as NF-κB and MAPK, and significantly relieve the symptoms of IBD [58]. The interaction between pro-inflammatory and anti-inflammatory signaling pathways determines the occurrence and development of IBD. Ginsenosides and their metabolites regulate a variety of inflammatory signaling pathways with their multitarget characteristics so as to effectively alleviate IBD.

## Conclusions

In summary, the potential protective effects of ginsenosides on IBD treatment are very definite and effective. They are the main active components of Panax ginseng. IBD is typically characterized by severe destruction of intestinal barrier function, including excessive apoptosis of IECs, excessive secretion of cytokines, and imbalance of immune status. Aminosalicylates, corticosteroids, immunosuppressive drugs, and monoclonal antibodies to TNF-α are well-established pharmacological therapies for IBD. However, these drugs have not always been effective against IBD and have some side effects. Recent studies focused on the effects of natural anti-inflammatory drug ginsenosides on IBD treatment, largely due to the safety, reliability, and affordability of ginsenosides in clinic. Ginsenosides are important in inhibiting TLR4/NF-κB/NLRP signal transduction, regulating inflammatory cytokine expression, and inducing immune cell maturation and differentiation to relieve the inflammatory injury in the colonic mucosa of patients with colitis. The effects of ginsenosides on immunoregulation and intestinal epithelial regeneration aim to enhance the intestinal mucosal barrier function. Hence, ginsenosides might serve as a promising new drug for treating IBD.

Ginsenosides should be developed and widely used in the future to alleviate inflammatory injury in the colonic mucosa of patients with IBD owing to its therapeutic effect, less side effects, and high acceptability.

## Abbreviations

AMPK: AMP-activated kinase
CD: Crohn’s disease
DSS: Dextran sulfate sodium
ERK: Extracellular regulated protein kinases
GC-TOFMS: Gas chromatography with time-of-flight mass spectrometry
HT-29: Colorectal cancer cells
IBD: Inflammatory bowel disease
IFN-γ: Interferon-γ
ILs: Interleukins
JNK: C-Jun n-terminal kinase
MAPK: Mitogen-activated protein kinases
NF-κB: Nuclear factor-kappa B
NLRP: Nucleotide-binding oligomerization domain-like receptor protein
NO: Nitric oxide
PU.1: Purine-rich nucleic acid-binding protein
TGF-β: Transforming growth factor-beta
TLR4: Toll-like receptor 4
TNBS: 2,4,6-Trinitrobenzene sulfonic acid
TNF-α: Tumor necrosis factor-α
UC: Ulcerative colitis
